# COVID-19-related end stage lung disease: two distinct phenotypes

**DOI:** 10.1080/07853890.2022.2039954

**Published:** 2022-02-16

**Authors:** Kostantinos Kostopanagiotou, Macé M. Schuurmans, Ilhan Inci, René Hage

**Affiliations:** a Department of Thoracic Surgery, University Hospital Zurich, Zurich, Switzerland; b Division of Pulmonology, University Hospital Zurich, Zurich, Switzerland; c Faculty of Medicine, University of Zurich, Zurich, Switzerland

**Keywords:** Pulmonary fibrosis, hypothesis, prognosis, lung transplantation

## Abstract

In COVID-19 related end stage lung disease, there are two distinct phenotypes. The first phenotype is the COVID-19 related acute respiratory distress syndrome (CARDS) showing a classical histopathological pattern of fibrotic diffuse alveolar damage (DAD). The second phenotype is the post-COVID pulmonary fibrosis (PCPF), in which the diagnosis is based on the combined clinical, radiological and (if available) pathological information. Both phenotypes have different clinical features, risk factors, biomarkers and pathophysiology. The exact prognosis in these two phenotypes as well as optimal treatment needs further studies.

**Key messages**
- Two different phenotypes exist for COVID-19 related pulmonary fibrosis. The CARDS phenotype has a worse prognosis compared to the PCPF phenotype, which requires longer-term follow-up and evolves without ARDS picture. The best treatment options for the two different phenotypes, such as anti-fibrotic drugs or lung transplantation, still needs to be defined in future studies.

Coronavirus Disease 2019 (COVID-19) either resolves spontaneously or presents with a 2% fatality rate. Severe COVID-19 due to massive alveolar damage with destruction of the pulmonary architecture, leads to restrictive ventilatory dysfunction, impaired gas exchange and severe respiratory failure.

Although many studies focus on the acute respiratory distress syndrome (ARDS), a recent article discussed two different phenotypes of lung fibrosis after COVID-19 [1]. The features or main characteristics of these two phenotypes known to date are compiled in Table 1.

**Table 1.** Different aspects of the two phenotypes of COVID associated lung fibrosis.

	COVID-19 related ARDS (CARDS)	post-COVID pulmonary fibrosis (PCPF)
Clinical features	7–14 days after initial symptoms secondary pulmonary hypertension +++	4–6 to 12 weeks after initial infection secondary pulmonary hypertension +
Mortality 90 days	30–50%	unknown
Risk factors	Mechanical ventilation, VILI, hyperoxia, prolonged hypoxia, increased BMI, elderly patients, possibly thromboembolism and hypercoagulability, possibly NETS	profound dyspnoea, higher respiratory rate, comorbid hypertension, ICU admission, hyperoxia, prolonged hypoxia, elderly patients, possibly thromboembolism and hypercoagulability, possibly NETS, higher CRP levels, lymphocytopenia, neutrophilia, eosinopenia lower baseline IFN-γ and MCP-3
Biomarkers	Classic “cytokine storm” not observed IL-6 moderately increased persistent deactivation of key immune cells e.g. reduced surface expression of the mHLA-DR	cytokine-driven: TGF-β and IL-1β longer telomere lengths appear to be protective, this genomic biomarker estimates balance of profibrotic and antifibrotic susceptibilities
Restrictive ventilatory defect	++	+++ (rib cage shrinkage)
Pneumothorax	+++	++
Pathophysiology	Severe pulmonary infiltration/edema and endothelitis	inflammation leading to impaired alveolar homeostasis, alteration of pulmonary physiology resulting in pulmonary fibrosis

ARDS = Acute Respiratory Distress Syndrome, CARDS = COVID-19 related ARDS, CRP = C-reactive protein, IFN-γ = interferon gamma, IL = interleukin, MCP-3 = monocyte chemoattractant protein 3, mHLA-DR = monocytic human leucocyte antigen-DR, NETS = neutrophil extracellular traps, PCPF = post-COVID pulmonary fibrosis, TGF = Tumour Growth Factor, VILI = mechanical ventilation-induced lung injury.

The first phenotype is the COVID-19 related ARDS (CARDS), characterized by the classical histopathological pattern of fibrotic diffuse alveolar damage or “fibrotic DAD” [2]. The first phase of DAD is the exudative phase in which plasma fibrin leaks into the interstitium and alveolar space where it polymerizes and creates hyaline membranes [2]. This is enhanced by strong alveolar inflammation caused by inflammatory cells infiltrating the alveoli [2].

The second phase is the proliferative phase, characterized by fibroblastic and myofibroblastic proliferation and extracellular matrix deposition resulting in fibrosis [2]. The squamous metaplasia further contributes to the fibrotic process [2].

Patients with CARDS typically require intubation, mechanical ventilation and/or extracorporeal membrane oxygenation (ECMO), or have previously undergone this treatment. Often these patients are overweight, which is associated with increased risk of developing ARDS for at-risk patients [3]. An increased body mass index is also a known risk factor for lung fibrosis. As the disease evolves, the bilateral ground glass opacities and consolidations rapidly increase leading to ARDS 3 weeks after the onset of symptoms. Many survivors of this acute phase of COVID-19 presenting as CARDS, especially those with a disease duration longer than 3 weeks, eventually develop fibrosis [4]. These fibrotic changes do not resolve within the first year after COVID-19 [3]. This progressive fibrosis can eventually be fatal, even in initial ARDS survivors.

The second phenotype in COVID-19 patients is the post-COVID pulmonary fibrosis (PCPF). The diagnosis of this fibrosis type, is based on the combined clinical, radiological and (if available) pathological information.

Clinically, the pulmonary function tests reveal a restrictive ventilatory defect in about 25% of patients with severe COVID-19 disease (WHO Severity Grade 3 and 4), and is associated with a significantly lower diffusing capacity in almost all patients 6 weeks after hospital discharge. Laboratory investigations in patients developing PCPF generally tend to have higher C-reactive protein (CRP) and d-dimer levels than in CARDS. Typically, decreased lymphocytes and eosinophils are noted, as well as elevated leukocytes and neutrophils.

The radiological features of PCPF include fibrotic changes, with reticular opacities and traction bronchiectasis with or without honeycombing although there are no strict criteria describing this type of pulmonary fibrosis yet, other than that the fibrosis occurred following COVID-19 diagnosis.

Chest computed tomography (CT) is an important diagnostic tool preferably used at an early stage. Histology is not only difficult to obtain, but also has no immediate therapeutic consequences. In addition, obtaining these biopsies surgically or bronchoscopically is a high-risk aerosolizing procedure.

Older age is a risk factor for PCPF [5], as well as for idiopathic pulmonary fibrosis (IPF) [6]. In elderly patients there is an accumulation of environmental factors, a weaker immune system, decreased stem cell stability, increased apoptosis and reactive oxygen species accumulation. These factors could lead to increased fibroblast proliferation, decreased alveolar and fibroblast stability, and an increased demand for proliferation, resulting in fibrosis [6]. Although speculative, this might explain the higher age as a risk factor for PCPF. In contrast to CARDS, the lung fibrosis of PCPF patients develops without previous ARDS, and can occur in all stages of COVID-19 severity.

In both phenotypes an important contributing iatrogenic component could be the prolonged high oxygen concentration treatment and potentially the barotrauma resulting in ventilator-induced lung injury. However, also spontaneously breathing patients can develop a severe and rapidly progressive PCPF [7].

Viral infections sometimes precede pulmonary fibrosis diagnosis [8], being a co-factor not a cause of IPF onset. PCPF may differ from IPF in terms of trigger event, pathophysiologic pathway and prognosis. Since PCPF is COVID-19-related, it is by definition not idiopathic. Moreover, PCPF can potentially be (partly) reversible [9].

In summary, COVID-19-related lung fibrosis has two different faces: CARDS with a dismal short-term prognosis and PCPF, which can be progressive after the acute phase of COVID-19. It might take several years to develop the full burden of PCPF patients and lung transplantation may be a treatment option for selected cases. For this reason, patients with discrete fibrotic changes after COVID-19 should be followed carefully and antifibrotic medication should be considered. Several clinical studies with either nintedanib (*n* = 4) or pirfenidone (*n* = 4) are currently underway (clinicaltrial.gov).

## Author contributions

M.S., K.K., I.I. and R.H. were involved in the conception and design, and the drafting of the paper, MS and RH were involved in revising it critically for intellectual content; and the final approval of the version to be published; and all authors agree to be accountable for all aspects of the work.
